# Establishment of the Maximum Residual Limit in the Milk of Dairy Cows Injected Intramuscularly with Prednisolone

**DOI:** 10.3390/vetsci10100614

**Published:** 2023-10-10

**Authors:** Seung Shik Rha, Ye Jin Yang, Woo Hyun Kim, Yeung Bae Jin, Kwang Il Park, Hu-Jang Lee

**Affiliations:** 1 Department of Environmental Health Sciences, Graduate School of Public Health, Seoul National University, Seoul 08826, Republic of Korea; james.rha@gcvp.co.kr; 2 College of Veterinary Medicine, Gyeongsang National University, Jinju 52828, Republic of Korea; yang93810@gnu.ac.kr (Y.J.Y.); woohyun.kim@gnu.ac.kr (W.H.K.); ybjin@gnu.ac.kr (Y.B.J.)

**Keywords:** bovine milk, LC-MS/MS, prednisolone, withdrawal time

## Abstract

**Simple Summary:**

Prednisolone (PSL) is generally used as an anti-inflammatory and analgesic agent. To establish a withdrawal period using PSL in this study, PSL residual levels and withdrawal time (WT) were assessed in milk after PSL was administered to healthy cows. As a result, the LC-MS/MS method’s recovery rate, relative standard deviation, and detection sensitivity met all required standards. At 24 h after treatment, the amount of PSL in milk containing 10 and 20 mg/mL of PSL was lower than the LOD and the LOQ. The time limit for withdrawal was set at 12 h. In summary, since the residual withdrawal period of PSL in milk is shorter than that of the test drug, it suggests that the administration of PSL preparations will produce less milk wastage than that of previous recommendations.

**Abstract:**

We measured the levels of prednisolone (PSL) residues in milk of intramuscularly dosed dairy cows and established a withdrawal time (WT) of PSL in milk. Eight healthy Holstein cows were injected with 10 (PSL-1) or 20 (PSL-2) mL of 10 mg/mL of PSL, and then, their milk was sampled at 12 h intervals for five days. PSL residue concentrations in milk were determined using LC-MS/MS. The correlation coefficient of the calibration curve was 0.9976. The limit of detection (LOD) and the limit of quantification (LOQ) were 0.2 µg/kg and 0.6 μg/kg, respectively. Recoveries ranged from 96.5% to 110.0%, and the coefficient of variation was <5.64%. At 24 h after administration, PSL levels in PSL-1 and PSL-2 were below the LOQ in all milk samples. Although this study had a smaller sample size than the European Medicines Agency’s recommendations (*n* = 20), it was based on the Animal and Plant Quarantine Agency guidelines of the Republic of Korea (*n* = 8) for the determination of withdrawal periods in milk. We established the withdrawal period for both PSL-1 and PSL-2 in milk at 12 h. In conclusion, we developed an analytical method that is sensitive and can reliably detect PSL in milk, and our estimated WT of PSL in bovine milk is shorter than the current 3-day withdrawal period of PSL in commercial PSL products.

## 1. Introduction

As the consumption of meat and meat products increases with rising populations and income levels, their quality has become just as important as their quantity [1]. Consequently, food safety is a main concern for consumers, policy makers, producers, and meat processors [2,3]. In general, antibiotics are used to improve livestock productivity, enhance feed efficiency, and prevent and treat livestock diseases [4,5]. Despite antibiotics having advantages, they pose a risk to human life due to their indiscriminate use, overdose, and misuse [4]. Antibiotics that are injected into livestock can remain in its edible parts, and this has been associated with the emergence of antibiotic-resistant bacteria [6]. In the Republic of Korea, the addition of antibiotics to the formulated feed has been banned since July 2011 [7], resulting in a decrease in the use of antibiotics in livestock. However, since 2016, for the purpose of preventing and treating livestock diseases, antibiotic usage has started to increase [1]. Several growth hormones used in livestock have high affinity to estrogen and androgen receptors; therefore, if they remain in meat and livestock products, they can, upon ingestion, affect growth, development, and reproduction by disrupting the action of hormones in the human body [8,9]. In addition, the consumption of milk and dairy products contaminated with animal growth hormones has been found to be a potential risk factor for various cancers [9]. Prednisolone (PSL) is a synthetic glucocorticoid commonly used as a potent anti-inflammatory, immunosuppressive, and analgesic agent for humans and animals. It has been used to treat mastitis, ketosis, musculoskeletal disorders, allergic diseases, and skin diseases in ruminants [10]. The use of PSL for growth promotion in livestock has been banned in many countries, and its use is permitted only for the treatment of cattle diseases [11]. Even when used to treat disease, the use of PSL is strictly controlled and can only be used if prescribed by a veterinarian. Also, PSL residues can be potentially toxic to consumers, so their use in livestock must be strictly regulated [11]. Therefore, establishing the withdrawal period of PLS is an essential issue, and it is strictly regulated by setting a maximum retention limit (MRL) for milk [11,12]. For animal medicines that have no set MRL values, the United States, the European Union, and Japan have introduced a Positive List System (PLS) that applies a uniform standard (0.01 mg/kg) to all animal medicines. For food safety, the distribution and sale of meat and its by-products that exceed this prescribed standard are prohibited [13].

In December 2020, the Korean government announced plans to implement, from 1 January 2024, PLS for fish, cattle, pigs, chicken, milk, eggs, etc., to prevent misuse and abuse of animal medicines and enhance the safety of imported livestock and fisheries products [14]. Accordingly, the Animal and Plant Quarantine Agency is in the process of resetting the withdrawal period for 180 product groups. Since 2020, these tests have been collecting data on drug residues from products exposed to any of 2500 veterinary drugs that are subject to PLS [15].

Because PSL injections lack domestic residual test data, the aim of this study was to determine the withdrawal period of PLS in milk of dairy cows that had been injected with this drug.

## 2. Materials and Methods

### 2.1. Reagents

The PSL used in our experiments was provided by the Daesung Microbiology Research Center (Uiwang, Korea). The PSL standard (purity, 98%), formic acid (purity, 95%), and acetonitrile (ACN) were purchased from Merck KGaA (Darmstadt, Germany). Other solvents used for our analysis were of HPLC grade and were purchased from Merck KGaA.

### 2.2. Animals

Sixteen healthy, lactating Holstein dairy cows raised on a dairy ranch near Jinju, Gyeongsangnam-do, Republic of Korea, were used for the experiment. Animals were stratified as follows: 4 high yield cows in early lactation stage, including cows with more than two births, and 4 low yield cows in late lactation stage according to Animal and Plant Quarantine Agency in Republic of Korea. All animal experiments were approved by the Institutional Animal Care and Use Committee (IACUC) of Gyeongsang National University (approval number GNU-201021-A0078) and performed in compliance with the guidelines of the IACUC of Gyeongsang National University, Republic of Korea.

### 2.3. PSL Administration and Sample Collection

The experimental cows were divided into two groups: the first group (PSL-1, *n* = 8) was intramuscularly injected once with the maximum recommended dose (10 mL/head of PSL at 100 mg/head) of the drug and the second group (PSL-2, *n* = 8) was administered twice with the maximum recommended dose (20 mL/head of PSL at 200 mg/head). Fifty milliliters of milk samples were collected and placed into 50 mL conical tubes at 0, 12, 24, 36, 48, 60, 72, 84, 96, 108, and 120 h after administering drugs to the dairy cows. Samples were stored in at −20 °C until they were analyzed.

### 2.4. Preparation of Standard Stock Solutions and Concentration Standards

One milligram of the PSL standard was precisely weighed on a balance and then placed into a 1000 mL quantitative flask to produce a 1.0 mg/L standard stock solution using methanol. The standard stock solution was then serially diluted using 10% ANC to produce standard solutions containing PSL at 1.0, 2.0, 4.0, 10, 20, 100, and 200 ng/mL concentrations. These standard solutions were stored at 4 °C until use.

### 2.5. Sample Pretreatment

According to the protocol in the Food Code [16], we placed 1 mL of homogenized sample into a 15 mL centrifuge tube and added 10 μL of cortisone (internal standard, 2 μg/mL). The mixture was shaken using a vortex mixer for 1 min, and then, 100 μL of a 20% trichloroacetic acid (TCA) solution was added. This mixture was shaken using a vortex mixer for 10 min. The protein and fat contents of this mixture were separated using centrifugation at 12,000× *g* rpm for 10 min. The resulting supernatant (500 µL) was collected and adsorbed by loading it onto a C_18_ cartridge (Waters, Framingham, MA, USA) activated with 5 mL each of water and methanol. C_18_ cartridge was washed first with 5 mL of water, followed by 5 mL of 20% aqueous acetone solution, and then 5 mL of n-hexane. After washing, C_18_ cartridge was eluted with 6 mL of ethyl acetate. The eluate was concentrated with nitrogen gas at 50 °C, and the residue was dissolved in 1 mL of 10% ACN. This solution was filtered through a 0.2 µm polytetrafluoroethylene membrane filter (Millipore Merck Korea, Seoul, Republic of Korea), and the filtrate was used as the test solution.

### 2.6. LC-MS/MS Conditions

PSL in milk samples was measured using liquid chromatography–electrospray tandem mass spectrometry (LC-MS/MS) (API4000, AB SCIEX, Concord, ON, Canada). The LC system consisted of an Agilent 1260 series machine (Agilent Technologies, Waldbronn, Germany) with an Atlantis dC_18_ Column (2.1 × 150 mm, 3.0 μm, Waters Corporation, Milford, MA, USA). The column temperature was maintained at 40 °C. The mobile phases A and B consisted of 0.1% formic acid (aqueous solution) and 0.1% formic acid (ACN solution), respectively. The flow rate was 0.3 mL/min, and the total running time on the chromatograph was 8 min. A triple quad 4500 system (Agilent Technologies, Waldbronn, Germany) was used for mass spectrometry. Electrospray ionization was used in the negative ion mode with the capillary voltage set to 3.0 kV. The multiple reaction monitoring transition for PSL was *m*/*z* 361.0 → 147.2.

### 2.7. Validation

The previously prepared standard solutions (1.0–200 ng/mL) were used to generate a calibration curve. The coefficient of correlation (R^2^) was obtained and was used to confirm the linearity of the calibration curve. The detection and quantification limits were determined according to the method described in a previous study [17]. A PSL standard solution was added to a blank so that the MRL in milk was 0.5 (3.0 ng/mL). This solution was then pretreated and analyzed according to the methods described above. On the chromatogram, PSL was calculated as 3 times and 10 times the concentration of the signal-to-noise ratio (S/N ratio), respectively. To measure the recovery rate, PSL was added to a blank sample (containing no PSL) to obtain final concentrations of 2.0, 20, and 200 ng/mL. The solutions were pretreated as described above. PSL recovery (%) was obtained from the peak area ratio of the extracted milk sample to the peak area of the PSL standard. This experiment was repeated three times, and to verify the precision of the recovery rate, relative standard deviations were obtained from the recovery rate of the third experiment. On the basis of the Ministry of Food and Drug Safety’s Guidelines for Analysis of Residual Animal Drugs [18], we considered half of the quantification limit residue as the tolerance standard required for analytical method verification; we considered a recovery rate of 60–120% and a relative standard deviation of 20–30% or less as the verification criteria for the measured limits of quantification, recoveries, and relative standard deviations.

### 2.8. Establishment of a Residual Withdrawal Period for PSL in Milk

On the basis of MRL (6.0 ng/mL) of PSL in milk, the European Medicines Agency’s (EMA) WT 1.4 program was used to set the withdrawal period. The appropriate withdrawal period was set by applying the 95% confidence level and the upper limit of the 99% tolerance [19]. In the case of samples containing PSL at below the quantification limit (3.0 ng/mL), half the value of the quantification limit was applied as the residual amount.

## 3. Results

### 3.1. Chromatogram of PSL

**Figure 1:**
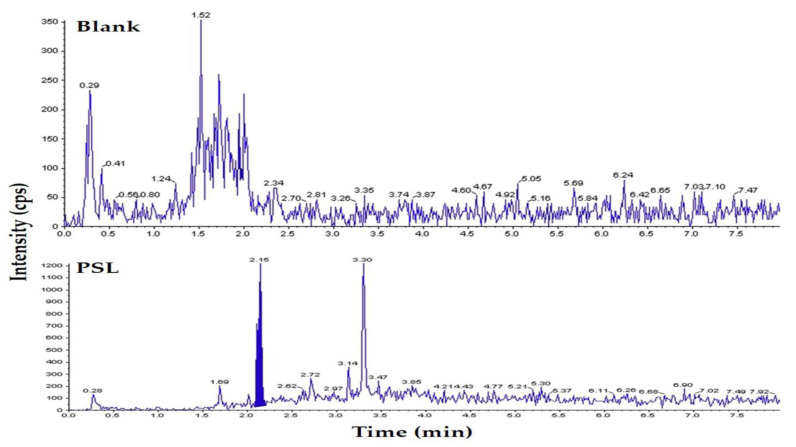
LC-MS/MS chromatogram of standard solution sample with prednisolone (PSL, 1.0 ng/mL).

Figure 1 shows a chromatogram generated using LC-MS/MS from the PSL standard solution (1.0 ng/mL). The retention time of PSL was 2.15 min, within a total run time of 8 min. In the negative ion mode, [M+H]-in *m*/*z* 361.0 was detected as the base ion, which was selected as the precursor ion. In the product ion scan, the *m*/*z* 147.2 ion appeared as a characteristic ion and was selected as a quaternary ion.

### 3.2. Calibration Curve and the Limits of Detection and Quantification

**Figure 2:**
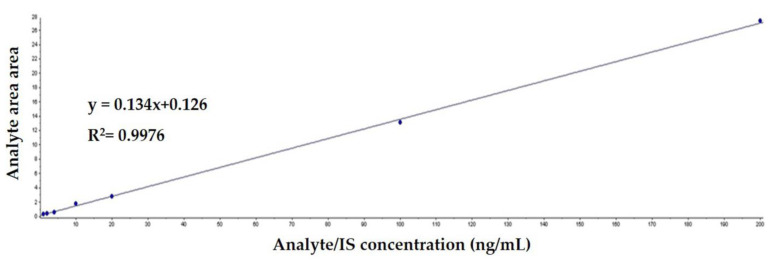
Calibration curve for prednisolone in milk.

LC-MS/MS analysis of standard PSL solutions at concentrations of 1.0, 2.0, 4.0, 10, 20, 100, and 220 ng/mL yielded a correlation coefficient of 0.9976, indicating a good linearity (Figure 2). In addition, the LOD and LOQ of LC-MS/MS were 0.6 ng/mL and 0.2 ng/mL, respectively.

### 3.3. Recovery Rate and Precision

The recovery rate and precision were obtained by repeatedly (three times) measuring the amount of PSL recovered after extracting and purifying it from milk samples with a known amount of PSL. The recovery rate and precision estimates are shown in Table 1. Recovery rates ranged from 96.5–110.0%, while the coefficient of variation ranged from 2.07–5.64%.

### 3.4. Analysis of PSL Residues in Milk

The amount of PSL residues in milk were determined by measuring the concentrations of PSL from PSL-1 and PSL-2 cows at various times after intramuscular administration of PSL. The results of the analysis are shown in Table 2. Twelve hours after administration of PSL in both groups of cows, PSL was below the LOQ in all milk samples collected from all eight cows.

### 3.5. PSL Residual Withdrawal Period Set in Milk

We attempted to use the WT 1.4 program to determine the withdrawal period of PSL in milk. We found that amounts of PSL residues in all samples were less than the LOQ, and therefore, the program could not be applied. In cases when the residual concentrations of a drug in all milk samples are below the LOQ (thus making it impossible to calculate a meaningful 95/95 acceptance limit), the EMA guideline stipulates that if the LOQ is sufficiently lower than or equal to the MRL, then the time when the first sample is collected after administration of the drug (i.e., 12 h) can be set as the withdrawal period [20]. Therefore, in this study, it is reasonable to set the withdrawal period of PSL in milk to 0.5 days for both PSL-1 and PSL-2.

## 4. Discussion

In this study, PSL was administered via intramuscular injection of a single dose of either 100 or 200 mg PSL per cow. Residual concentrations of PSL in milk samples collected after different time periods were analyzed using LC-MS/MS, and then, the appropriate withdrawal period of PSL was calculated.

Previous studies that analyzed PSL residues in milk samples, using LC-MS/MS, found the following retention times for the PSL peak in their respective chromatograms: 7.34 [17], 1.65 [21], and 12.97 [22] min. In this study, the retention time of PSL was 2.15 min, which is shorter than those described in two previous studies [17,22], but longer than that reported in another study [21]. Retention time depends on the conditions of the analytical instrument used, as well as the sample to be analyzed. It is thought that there is a difference in the retention time of the chromatogram peak of PSL. The analysis time (retention time, 4.5 min) for the PSL residue analysis method established in this study is shorter than that reported in the Food Code [16]. Thus, the analysis efficiency was increased.

Previous studies that analyzed PSL in milk reported correlation coefficients of the calibration curves of PSL of greater than 0.998, which is slightly higher than that obtained in this study (0.9976) [17,21,22]. Nevertheless, all values show high linearity and satisfy the Codex [23], which recommends a correlation coefficient of 0.98 or higher.

The “Standards for residues of veterinary drugs in food” of the Korea Food Code [24] has set the MRL of PSL in milk at 6.0 ng/kg. On the basis of our analysis, the PSL residues in milk are at levels that are possibly <1/10 the MRL value.

The Practical Guidelines for Animal Drug Residue Analysis Method of the Ministry of Food and Drug Safety [18] stipulates that if the criterion for accepting drug residue levels in food is less than 0.1 mg/kg, then the limit of quantification should be less than 0.05 mg/kg. Therefore, the limits of detection (0.2 ng/mL) and quantification (0.6 ng/mL) established in this study meet the analysis criteria [18] of the Practical Guidelines for Analysis of Residual Veterinary Drugs. In a study that established a residue analysis method for detecting PSL in milk using LC-MS/MS, Lee et al. reported that the detection and quantification limits for PSL were 4.60 and 11.46 ng/mL, respectively [17]. Previous study that reported that the limits of detection and quantification were 18 and 40 ng/mL, respectively [21]. Another study simultaneously analyzed 12 corticosteroids in milk using LC-MS/MS and found that the limits of detection and quantification of PSL were 0.009 and 0.03 ng/mL, respectively. Therefore, the limits of detection and quantification in the present study were both lower [17,21] and higher than [21] those reported in previous studies. The reason for this result may be the differences in sample pre-processing methods and analysis equipment used in the different studies.

As verification standards, the Practical Guidelines for Animal Drug Residue Analysis Method of the Ministry of Food and Drug Safety [18] states that the recovery rate of drugs in food should be within 80–110%, and the coefficient of variation should be less than 15% at a concentration of 0.1 mg/kg or more. In this study, the recovery rate and coefficient of variation of PSL were 96.5–110.0% and 2.07–5.64%, respectively. Therefore, our analytical method meets the verification criteria required for analytical method verification [18]. The recovery rate and coefficient of variation of PSL in milk ranged from 70.9–101.7% and 2.39–8.67%, respectively, in a study by Lee et al. and Kaufmann et al. reported ranges of 98.3–109.4% and 2.9–14.5%, respectively [17,25], and Cui et al. reported ranges of 110.7–114.9% and 8.0–9.3%, respectively [26]. The recovery rate of this study is slightly higher [17,25] and slightly lower [26] than these previously reported rates. Our coefficient of variation is lower than these previously reported coefficients.

In a previous study, 11 mg of PSL was injected into the udder of a cow twice within a 24 h interval. Residual concentrations of PSL in milk samples collected at 12 and 24 h were 0.81–235 ng/mL and 0.81–4.3 ng/mL, respectively [27]. In a study on mastitis-infected cows [28], 10 mg of PSL was injected into a cow’s udder three times at 24 h intervals, resulting in a PSL milk concentration of 110 ng/mL at 12 h. Similarly, in a study on healthy cows not infected with mastitis [29], 10 mg of PSL was injected into a cow’s udder three times at 24 h intervals, resulting in a PSL milk concentration of 1.1 ng/mL at 12 h. In this study, 100 mg and 200 mg of PSL were administered intramuscularly to milking cows once, resulting in residual PSL concentrations in milk below the LOQ (0.2 ng/mL) at 12 h after administration. Compared to previous research results, the residual concentration of PSL in milk was very low, which may be due to differences in the administration dose and route of administration of PSL. It is believed that PSL injected directly into the udder of milking cows results in higher residual concentration of PSL in milk.

In this study, PSL was administered at 100 and 200 mg per cow, and for PSL residual analysis in milk, the withdrawal period was set as 0.5 days according to the EMA guidelines for withdrawal period establishment in milk [19]. The MSD Animal Health at Rahway, NJ, USA, administers PSL using the same ingredients and dosage as those used in this study (i.e., 100–200 mg PSL/head). This case has set the withdrawal period for PSL in milk at 3 days [30]. Meanwhile, the animal drug management system of Agriculture, Forestry and Livestock Quarantine Headquarters [2] recommends a maximum PSL dose of 100 mg/head. Using this dosage, the residual withdrawal period of PSL in milk was set at 3 days. Our experiments yielded a much shorter residual withdrawal period of PSL in milk, which may be due to the weight range of milking cows.

This study was conducted following the guidelines of the Animal and Plant Quarantine Agency in Republic of Korea. It used a smaller sample size than the recommendations of the “Guideline on determination of withdrawal periods for milk” [19] of the European Medicines Agency (*n* = 20).

## 5. Conclusions

The recovery rate, relative standard deviation, and detection sensitivity of the LC-MS/MS method for PSL in milk satisfies all the standards of the Ministry of Food and Drug Safety’s method for analyzing residual veterinary drugs. Moreover, we determined that the residual withdrawal period of PSL in milk is shorter than that of the test drug. Thus, the administration of PSL preparations could imply less milk wastage than previous recommendations.

## Figures and Tables

**Table 1:** Recovery and precision of prednisolone spiked into milk.

Concentration (ng/mL)	Recovery (%)			Recovery (%)	CV (%)
	No. 1	No. 2	No. 3		
2.0	106.5	111.0	108.0	108.5 ± 2.3	2.12
20	101.0	113.0	109.5	110.0 ± 6.2	5.64
100	94.7	96.2	98.7	96.5 ± 2.0	2.07

CV (Coefficient of variation) = (Standard deviation/Mean) × 100.

**Table 2:** Prednisolone concentration in milk of treatment groups on day before treatment and at 12, 24, 36, 48, 60, 72, 84, 96, 108, and 120 h after treatment of test product via intramuscular injection.

Group	No.	Concentration (µg/kg)										
		Time after Treatment (h)										
		0 *	12	24	36	48	60	72	84	96	108	120
PSL-1	1	ND	<LOQ	<LOQ	<LOQ	<LOQ	<LOQ	<LOQ	<LOQ	<LOQ	<LOQ	<LOQ
	2	ND	<LOQ	<LOQ	<LOQ	<LOQ	<LOQ	<LOQ	<LOQ	<LOQ	<LOQ	<LOQ
	3	ND	<LOQ	<LOQ	<LOQ	<LOQ	<LOQ	<LOQ	<LOQ	<LOQ	<LOQ	<LOQ
	4	ND	<LOQ	<LOQ	<LOQ	<LOQ	<LOQ	<LOQ	<LOQ	<LOQ	<LOQ	<LOQ
	5	ND	<LOQ	<LOQ	<LOQ	<LOQ	<LOQ	<LOQ	<LOQ	<LOQ	<LOQ	<LOQ
	6	ND	<LOQ	<LOQ	<LOQ	<LOQ	<LOQ	<LOQ	<LOQ	<LOQ	<LOQ	<LOQ
	7	ND	<LOQ	<LOQ	<LOQ	<LOQ	<LOQ	<LOQ	<LOQ	<LOQ	<LOQ	<LOQ
	8	ND	<LOQ	<LOQ	<LOQ	<LOQ	<LOQ	<LOQ	<LOQ	<LOQ	<LOQ	<LOQ
	M ± SD	ND	<LOQ	<LOQ	<LOQ	<LOQ	<LOQ	<LOQ	<LOQ	<LOQ	<LOQ	<LOQ
PSL-2	1	ND	<LOQ	<LOQ	<LOQ	<LOQ	<LOQ	<LOQ	<LOQ	<LOQ	<LOQ	<LOQ
	2	ND	<LOQ	<LOQ	<LOQ	<LOQ	<LOQ	<LOQ	<LOQ	<LOQ	<LOQ	<LOQ
	3	ND	<LOQ	<LOQ	<LOQ	<LOQ	<LOQ	<LOQ	<LOQ	<LOQ	<LOQ	<LOQ
	4	ND	<LOQ	<LOQ	<LOQ	<LOQ	<LOQ	<LOQ	<LOQ	<LOQ	<LOQ	<LOQ
	5	ND	<LOQ	<LOQ	<LOQ	<LOQ	<LOQ	<LOQ	<LOQ	<LOQ	<LOQ	<LOQ
	6	ND	<LOQ	<LOQ	<LOQ	<LOQ	<LOQ	<LOQ	<LOQ	<LOQ	<LOQ	<LOQ
	7	ND	<LOQ	<LOQ	<LOQ	<LOQ	<LOQ	<LOQ	<LOQ	<LOQ	<LOQ	<LOQ
	8	ND	<LOQ	<LOQ	<LOQ	<LOQ	<LOQ	<LOQ	<LOQ	<LOQ	<LOQ	<LOQ
	M ± SD	ND	<LOQ	<LOQ	<LOQ	<LOQ	<LOQ	<LOQ	<LOQ	<LOQ	<LOQ	<LOQ

* Before treatment of test product. ND, not detected; LOQ, limit of quantitation; M ± SD, mean ± SD. LOQ of prednisolone in milk is 6.0 ng/mL. PSL-1, intramuscularly injected with prednisolone 100 mg/cow; PSL-2, intramuscularly injected with prednisolone 200 mg/cow.

## Data Availability

The data presented in this study are available upon request from the corresponding author.
